# Relationship Between Carotid Intima-Media Thickness with some Inflammatory Biomarkers, Ghrelin and Adiponectin in Iranians with and without Metabolic Syndrome in Isfahan Cohort Study

**Published:** 2010

**Authors:** Taiebeh Hajmohammadi, Masoumeh Sadeghi, Masoumeh Dashti, Mohammad Hashemi, Mohammad Saadatnia, Mojgan Soghrati, Mohammad Talaei, Nizal Sarrafzadegan

**Affiliations:** 1 Resident of Cardiology, Isfahan University of Medical Sciences; 2 Associate Professor of Cardiology, Isfahan Cardiovascular Research Center, Isfahan University of Medical Sciences; 3 Resident of Neurology, Isfahan University of Medical Sciences; 4 Associate Professor of Cardiology, Isfahan University of Medical Sciences; 5 Associate Professor of Neurology, Isfahan University of Medical Sciences; 6 General Practitoner, Medical Researcher; 7 Medical doctor, Isfahan Cardiovascular Research Center, Isfahan University of Medical Sciences; 8 Professor of Cardiology, Head of Isfahan Cardiovascular Research Center, Isfahan

**Keywords:** Intima-media thickness (IMT), Carotid artery, hs-CRP, Ghrelin, Adiponectin IL-6, IL-10

## Abstract

**BACKGROUND:**

Recent studies have confirmed inflammatory factors and metabolic syndrome (MetS) as important cardiovascular disease (CVD) risk factors. Recently measurement of carotid intima-media thickness (IMT) has been used for evaluation of early atherosclerosis. This study was designed to assess the correlation between IMT with some inflammatory biomarkers, ghrelin and adiponectin in people with and without MetS in a cohort sample in Isfahan province.

**METHODS:**

Among participants of Isfahan Cohort Study (ICS) by random sampling, 88 participants were selected and divided into case (with MetS) and control (without MetS) groups. A questionnaire including demographic data and CVD risk factors was completed for all of the participants. Physical examination and blood pressure, height, weight and waist circumference measurements were done for all subjects. Vascular echocardiography was done for evaluation of IMT of each carotid artery of both sides. Interlukin-6 (IL-6), interlukin-10 (IL-10), highly sensitive C-reactive protein (hs-CRP), ghrelin and adiponectin levels were measured using ELIZA method. Data were entered in SPSS_15_ software and analyzed by t-test, chi square, Pearson correlation and linear regression analyze.

**RESULTS:**

The mean waist circumference, BMI, systolic blood pressure, diastolic blood pressure, hs-CRP and IMT of left carotid artery were significantly higher in participants with Mets. There was significant correlation between left carotid IMT and IL-6 level in all patients (P = 0.03). After adjustment for age and sex, significant relationship in groups with MetS was only reported between the left IMT and IL-6 (P = 0.02). There was no relation between IMT and other inflammatory markers in subjects with and without MetS.

**CONCLUSION:**

Significant correlation between IL-6 and IMT was reported in patients with MetS. While no significant correlation between IL-10, adiponectin and ghrelin with IMT was observed in metabolic syndrome group.

## Introduction

Metabolic syndrome (MetS) is defined as the accumulation of several cardiovascular risk factors (CVD) in an individual including visceral obesity, hypertension, high triglyceride (TG) level, low level of high-density-lipoprotein cholesterol (HDL-C) and impaired glucose tolerance test.^1^ The prevalence of MetS in Iran has been estimated as 23.3% which is higher than some of the western countries^2^ and based on increasing prevalence of obesity in children and adolescents in Iran, prevalence of MetS will be increased in the future.^3^ Nowadays, despite various preventive modalities for CVD, it is still increasing, so screening for high risk individuals and using preventive methods is very important.^4^

Beside main risk factors of CVD like hypertension, diabetes, smoking and hyperlipidemia, recent studies have confirmed inflammatory factors and metabolic syndrome as important CVD risk factors.^5^, ^6^ Increase in C-reactive protein (CRP) level which is an inflammatory marker, is an independent predictor of CVD and an important factor in progression of metabolic syndrome.^4^–^6^ Correlation between CRP and subclinical atherosclerosis is not fully understood but two studies reported positive relationship between CRP and carotid plaque and another study has shown that correlation between CRP and carotid plaque depends on the degree of stenosis and severity of atherosclerosis.^7^, ^8^ Makita et al study showed significant correlation between CRP and carotid plaque only in men.^8^ Studies showed that the serum level of two inflammatory factors, interleukin 6 and interleukin 10, are related to the increase in prevalence of CVD.^5^, ^9^ Also, lower serum level of adiponectin (a specific obesity factor) has been suggested as an independent risk factor for CVD.^10^ Ghrelin is a marker of endothelial function which produces nitric oxide (NO) in the endothelial cells of the vessels. Lower serum levels of ghrelin have significant relationship with insulin resistance, type II diabetes and hypertension that leads to increase in atherosclerosis.^11^, ^12^ Few studies have been done on the effect of ghrelin on atherosclerosis in MetS and some of the studies have reported the reverse correlation between ghrelin and atherosclerosis.^13^, ^14^ Recently, measurement of carotid intima-media thickness (IMT) using ultrasonography as a non-invasive method has been used for evaluation of vascular damage but few studies have assessed the effect of metabolic syndrome on carotid artery IMT.^15^ It seems that assessment of the relationship between carotid IMT and some inflammatory factors, ghrelin and adiponectin in MetS, may predict asymptomatic vascular changes in patients with MetS. This study was designed to assess the correlation between carotid IMT with some inflammatory biomarkers, ghrelin and adiponectin in people with and without MetS in sample of cohort in Isfahan province.

## Materials and methods

This study was a done on a sample of Isfahan Cohort Study (ICS) participants. ICS has been started since 8 years ago (2001) as a part of baseline study of Isfahan Healthy Heart Program (IHHP).^16^ Participants ≥ 35 years that were selected via multi-stage cluster sampling from phase I of IHHP were included in ICS. Demographic and behavioral characteristics, blood pressure, body mass index(BMI) and routine laboratory tests like fasting blood sugar (FBS), 2 hour postprandial sugar, total cholesterol, LDL-cholesterol (LDL-C), HDL-cholesterol (HDL-C), triglyceride (TG) and electrocardiography for all of the participants were done and recorded at the first year of ICS. Then, every 2 years, each of the participants was followed by phone for assessment of primary outcomes of this cohort that included fatal or non-fatal myocardial infarction, fatal or non-fatal stroke, sudden cardiac death and hospitalization. In 2007, the tests and measurements were repeated again.^17^ Among 599 (39.4%) ICS participants in Isfahan (rural and urban) who suffered from MetS and remained in the study (not missed the follow-ups), 350 (58.4%) patients were randomly selected. The same number of subjects were selected from the rest of population (921) who did not have MetS (38%). Fifteen participants had died since the last follow up. On the whole, 472 (68.9%) attended the examinations. IMT test and laboratory measurements were carried out for a randomly selected sub-sample of this population consisted of 44 patients with MetS and 44 participants without it. Random selection procedures were done via random sampling (Sampling Wizard) in SPSS_15_ software. According to ATPIII criteria, metabolic syndrome is defined as having at least three of the five criteria: 1- waist circumference>102cm in men and >88 cm in women, 2- serum triglyceride>150mg/dl, 3- HDL<40 mg/dl in men and<50mg/dl in women, 4- FBS>110 mg/dl, 5- Blood pressure>130/85 mmHg.^18^ Inclusion criteria were the followings: participating in ICS from 2001 with ATPIII criteria for Mets.^18^ Exclusion criteria were the followings: participant's dissatisfaction, history of stroke or other cerebrovascular problems, pregnancy, history of carotid operation or carotid stent. The case and control group's questionnaires were assessed and patients who didn't have exclusion criteria were invited via telephone call. Written informed consent was obtained from all participants. A questionnaire including demographic data, CVD risk factors, history of diabetes, hypertension, smoking, hyperlipidemia and duration of these disease and all of the previous treatments, was completed for all participants in both case and control groups. Physical examination including blood pressure, height, weight and waist circumference measurements was done for all subjects using standard methods.^19^ Ten milliliter of fasting blood was obtained from the participants and sent to the laboratory of Isfahan Cardiovascular Research Center and after centrifuge, was preserved at -70 degrees of centigrade for necessary laboratory tests (ghrelin, interleukin 6 and 10, adiponectin and hs-CRP). Ghrelin was measured using ELISA method and BioVendor kits. Then, all subjects in both case and control groups underwent vascular echocardiography for evaluation of carotid IMT. Carotid IMT was measured by a neurologist using Vivid-3 echocardiograph (Vivid 3-Japan) in both carotid arteries in three points: 1- terminal 2 cm of common carotid artery before the division of carotid flow, 2- carotid bulb in 2 cm from the part of carotid division, 3- internal carotid artery, 2 cm after carotid division. Normal carotid IMT is<0.9 mm and between 0.9-1.5 mm is considered abnormal. IL-6 and IL-10 were measured via ELIZA method using Medsystem kit (Bendermed-Austria). Adiponectin and ghrelin were measured via ELIZA method, using Biovendor kit (Germany) and hs-CRP was measured via immunoturbidimetry method with Autoanalyzer (Hitachi 902) with Pars Azmun kit (Iran). Data were analyzed via SPSS_15_ software with t-test, ANOVA and chi-Square for comparison between the two groups and linear regression models and Pearson correlation for finding relation between variables.

## Results

Eighty eight subjects were enrolled in this study and were divided into case and control groups with 44 subjects in each group. Clinical and biological characteristics of the study subjects are summarized in Table 1. The mean waist circumference (P = 0.001), BMI (P = 0.00), systolic blood pressure (P = 0.001), diastolic blood pressure (P = 0.001), hs-CRP (P = 0.006) and IMT of left carotid artery (P = 0.02) were significantly higher in participants with metabolic syndrome (case group, Table 1). Subjects with metabolic syndrome had significantly higher percentage of non-smokers or previously smokers compared with the control group (P = 0.02). No significant difference was reported between the two groups in mean age, FBS, total cholesterol, LDL-C, HDL-C, interlukin-6, interlukin-10, adiponectin, ghrelin and IMT of right carotid artery (Table 1). As it is shown in table 2, there was a significant correlation between mean left carotid IMT and IL-6 level in all patients (P = 0.035) and there was not any correlation between IMT and IL-10, adiponectin, ghrelin and hs-CRP. As it has been shown in table 3, after adjustment for age and sex, significant relationship in groups with MetS was only reported between left IMT and IL-6 (P = 0.02).

**Table 1:** Baseline clinical and laboratory characteristics of participants with or without metabolic syndrome.

Demographic Characteristics	Subjects with metabolic syndrome	Subjects without metabolic syndrome	P value
Age (years)	55.63±8.6	52.86±7.6	0.11
Smoking status (n.%)			
Never and past		93.2	75
Current	6.8	25	0.02
Male sex (n,%)	15(34.1%)	29(65.9%)	0.003
BMI (Kg/m^2^)	30.71±6.2	26.00±3.7	0.000
IL-6(pg/ml)	0.90±0.69	0.76±0.49	0.29
IL-10 (pg/ml)	1.06±1.1	1.28±1.2	0.40
Adiponectin (µg/ml)	14.25±10	14.46±10	0.92
Ghrelin (µg/ml)	106.53±66	135.24 ±84	0.09
hs-CRP (µg/ml)	20.12±25	7.70±10	0.006
Mean IMT. Rt (mm)	0.73±0.14	0.68±0.12	0.09
Mean IMT. Lt (mm)	0.77±0.19	0.68±0.14	0.02

Values are mean (SD). BMI: body mass index,IL-6:interleukin 6,IL-10:interleukin 10,hs-CRP:high sensitive CRP, IMT: intima-media thickness.

**Table 2:** Relation between mean IMT and inflammatory markers in all subjects.

	IL- 6		IL- 10		Adiponectin		Ghrelin		hs- CRP	
	
	B	P	B	P	B	P	B	P	B	P
Mean IMT. Rt	−0.12	0.25	0.038	0.72	−0.07	0.46	−0.029	0.78	−0.21	0.07
Mean IMT. Lt	−0.19	0.035	0.20	0.058	−0.16	0.08	−0.15	0.09	−0.14	0.17

Adjusted by age, sex, Mets.

**Table 3:** Relation between IMT and inflammatory markers in participants with and without metabolic syndrome.

Groups		IL-6		IL-10		Adiponectin		Ghrelin		hs-CRP	

		B coefficient	p	B coefficient	p	B coefficient	p	B coefficient	p	B coefficient	p
Mean IMT.Rt	MetS(+)	−0.234	0.12	−0.149	0.29	−0.149	0.29	−0.307	0.26	−0.123	0.43
	MetS(−)	0.140	0.40	0.011	0.94	0.011	0.94	0.252	0.09	−0.36	0.02
Mean IMT.Lt	MetS(+)	−0.249	0.04	−0.113	0.36	−0.113	0.36	−0.193	0.12	−0.071	0.61
	MetS(−)	0.057	0.72	−0.227	0.12	−0.227	0.12	−0.159	0.28	−0.258	0.09

*All data adjusted by sex and age

## Discussion

Results of the current study showed significant correlation between metabolic syndrome and female sex, BMI, hs-CRP and left carotid artery IMT (P<0.05). Although our results showed significant relationship between IL-6 level and right carotid artery IMT in patients with MetS, in this study IMT showed significant correlation with hs-CRP level in patients without MetS too. No significant correlation between IL-10, adiponectin and ghrelin with IMT in metabolic syndrome was observed in this study. The correlation between CRP and subclinical atherosclerosis isn't fully understood yet but Willeit et al and Heinrich et al reported positive relationship between CRP and the presence of carotid plaques. Willeit et al study showed that this correlation depends on the degree of stenosis and the severity of atherosclerosis.^20^, ^21^ In Willeit et al study, significant relationship was seenbetween CRP level with early nonstenotic atherosclerosis (equal or less than 40% narrowing of the lumen) not with advanced stenotic atherosclerosis (more than 40% narrowing of the lumen).^21^ Makita et al^8^ and Chen et al^4^ showed significant correlation between CRP and carotid plaque only in men. Elevated CRP level has been found in metabolic syndrome.^22^ In our study, although the relationship between CRP and Mets was significant, its relationship with IMT in Mets participants wasn't significant and CRP had significant relationship with IMT in participants without Mets which is different from the results of several studies that have reported significant correlation between hs-CRP and IMT in MetS.^4^ The absence of correlation between CRP and IMT in MetS participants in our study may be due to higher frequency of MetS in female sex compared with Makita et al^8^ and Chen et al^4^ studies which reported significant correlation between CRP and carotid plaque, only in men. Even more, our subjects had advanced stenotic atherosclerosis (more than 40% narrowing of the lumen) and according to Willeit et al study, advanced stenotic atherosclerosis has no correlation with CRP level.^21^ Tracy et al^7^ and Nishida et al^5^ did not find any correlation between CRP and IMT. Tataru et al didn't report any relationship between CRP and nonstenotic plaques (<50%) in patients with coronary heart disease^23^ Our results are close to Tracy et al^7^ and Nishida et al^5^ studies but different than Tataru et al^23^ study. This may be explained by the presence of advanced stenotic lesion in our subjects. We reported significant relationship between IL-6 and right carotid artery IMT in patients with MetS (P = 0.004). Nishida et al showed significant relationship between IL-6 and IMT only in men with MetS.^5^ We could not find any previous study on the relationship between IL-10 and IMT in MetS. In our study, no correlation between IMT and adiponectin was observed which is different than Nishida et al study that has shown negative correlation between IMT and adiponectin in men;^5^ this difference may be due to inclusion of more women with metabolic syndrome in our study sample. Dullaart et al study showed significant relationship between adiponectin and IMT too.^24^ We didn't find any significant positive or negative relationship between IMT and ghrelin in patients with metabolic syndrome. Ukkola et al reported positive correlation between ghrelin and IMT in Mets(P<0.01).^25^ Some studies have reported reverse correlation between ghrelin and atherosclerosis.^13^, ^14^ In summary, this study showed significant relationship between IL-6 and right coronary artery IMT in patients with MetS and significant correlation between IMT and hs-CRP level in patients without metabolic syndrome. No significant correlation between IL-10, adiponectin and ghrelin with IMT in MetS was observed in this study. The limitations of the present study included the small sample size and its design as a cross-sectional study which didn't enable any casual relationship to be established. Further investigations with large sample size and longer duration especially on ghrelin, adiponectin and IL-10 are necessary.
